# PEDF and Its Role in Metabolic Disease, Angiogenesis, Cardiovascular Disease, and Diabetes

**DOI:** 10.3390/biomedicines13071780

**Published:** 2025-07-21

**Authors:** Crispin R. Dass

**Affiliations:** 1 Curtin Medical School, Curtin University, Bentley 6102, Australia; crispin.dass@curtin.edu.au; Tel.: +61-8-9266-1489; 2 Curtin Medical Research Institute, Curtin University, Bentley 6102, Australia; 3 Faculty of Pharmacy, Silpakorn University, Nakhon Pathom 73000, Thailand

**Keywords:** PEDF, angiogenesis, cardiovascular disease, diabetes, metabolism

## Abstract

This review highlights recent findings on the potent anti-angiogenic serpin protein, pigment epithelium-derived factor (PEDF) as it relates to metabolic disease, diabetes, angiogenesis and cardiovascular disease (CVD), listing a majority of all the publicly available studies reported to date. PEDF is involved in various physiological roles in the body, and when awry, it triggers various disease states clinically. Biomarkers such as insulin, AMP-activated protein kinase alpha (AMPK-α), and peroxisome proliferator-activated receptor gamma (PPAR-γ) are involved in PEDF effects on metabolism. Wnt, insulin receptor substate (IRS), Akt, extracellular signal-regulated kinase (ERK), and mitogen-activated protein kinase (MAPK) are implicated in diabetes effects displayed by PEDF. For CVD, oxidised LDL, Wnt/β-catenin, and reactive oxygen species (ROS) are players intertwined with PEDF activity. The review also presents an outlook on where efforts could be devoted to bring this serpin closer to clinical trials for these diseases and others in general.

## 1. Introduction

The serpin family consists of two cohorts of proteins, The first comprises the predominant family of mammalian protease inhibitors, coordinating cascades such as inflammation, blood coagulation, and extracellular matrix (ECM) remodelling. The second encompasses a substantial number of serpins that, though structurally similar, are not thought to be inhibitors of specific proteases, but have important functions in a wide range of cellular processes. Pigment epithelium-derived factor (PEDF) is a member of the second group of serpins, but may regulate inflammation and ECM remodelling, so it traverses both categories. PEDF is a 418-amino-acid-long protein, with a versatile range of functions. As the most potent anti-angiogenic protein, and with roles that are diverse and potentially therapeutic, the significance of focusing on this protein for research is very high. These roles will be discussed in turn below, with recent updates to formulate a better understanding.

The PEDF gene is believed to have first appeared in vertebrates [1]. The PEDF gene is well-conserved amongst vertebrates, ranging from 48.3 kb in Xenopus tropicalis to 2.9 kb in fugu, with humans coming in at 15.6 kb [1]. The human gene encoding PEDF is located on chromosome 17p13.3 and comprises 9 exons encoding a 418-amino-acid protein (NCBI Reference Sequence: NG_028180.1). PEDF, a member of the serine protease inhibitor superfamily (SERPIN), has a reactive centre loop [2]. The secondary structure consists of three β-sheets and ten α-helices [3]. Most serpin members are serine protease inhibitors, which include antichymotrypsin and antitrypsin [4]. PEDF is considered to be a noninhibitory serpin however, as it is activated after chymotrypsin RCL cleavage, but importantly lacks protease activity [5]. For further information on PEDF background, the reader is referred to several recent comprehensive reviews written on the topic [6,7].

## 2. PEDF and Physiological Angiogenesis

PEDF is known to have a strong link with angiogenesis, and this link spans various physiological processes and ailments, as will be discussed below. Interaction between PEDF and vascular endothelial growth factor receptor 1 (VEGFR-1) or VEGFR-2 may be a possible mechanism for inhibiting angiogenesis [8]. It has been proposed that PEDF may be binding to the VEGF receptors to promote their internalisation and/or degradation, as a means to limit VEGF signalling in cells. PEDF KO pulmonary endothelial cells (ECs) proliferate at a slower rate and express increased levels of proinflammatory markers, for example VEGF, inducible nitric oxide synthase (iNOS), vascular cell adhesion molecule-1 (VCAM-1), as well as attenuated cellular junctional organisation, and nuclear localisation of β-catenin [9]. The lungs of PEDF KO mice exhibited increased expression of macrophage marker F4/80, along with increased thickness of the vascular walls, consistent with a proinflammatory phenotype.

Acute respiratory distress syndromes are acute injuries to pulmonary capillary ECs and alveolar epithelial cells caused by events such as severe infection and shock [10]. Differences were observed in the levels of VEGF and PEDF between the two groups, and the VEGF/PEDF ratio was strongly associated with the risk of death [11].

In retinal ECs isolated from PEDF KO mice, attenuation of capillary morphogenesis was noted, and there were increased levels of oxidative stress [12]. Wounds from PEDF KO mice had increased capillary formation during repair, and a delay in capillary pruning [13]. Conversely, exogenous PEDF decreased capillary density within skin wounds in wildtype mice. In vitro, PEDF inhibited migration and dermal microvascular ECs, causing a decrease in the expression of VEGFR2, VCAM-1, and other surface receptors.

Systemic sclerosis (SSc) is an autoimmune connective tissue disorder where fibrosis of the skin and vascular abnormalities within internal organs are noted. The VEGF/PEDF ratio significantly increases in progressive SSc patients [14], suggesting that it might be used as a biomarker of disease progression. One hallmark of SSc is also prominent fibrosis of the skin, and KO of PEDF in mice supports its role as an antifibrotic factor [15]. PEDF KO mice exhibit stromal overgrowth in several organs, including the pancreas and liver [16]. Previous studies have shown that PEDF is highly expressed in pulmonary fibrosis, suggesting a potential link of this factor with the pathogenesis of tissue fibrosis [17]. It has been shown that transforming growth factor β (TGF-β) suppresses angiogenesis in SSc through decreased fibroblast caveolin-1 expression and increased PEDF expression [18].

While the aetiology and pathogenesis of psoriasis are largely unknown, it is believed that angiogenesis plays a prominent role in the early stage of psoriasis and is also significantly involved in its recurrence [19]. Calcipotriol, a derivative of vitamin D3, is a first-line therapeutic against psoriasis and inhibits psoriasis-like angiogenic features by suppressing VEGF, increasing PEDF and decreasing microvessel density (MVD) [20].

A disintegrin and metalloproteinase with thrombospondin motifs 5 (ADAMTS5) expression is increased in the vitreous humour of proliferative diabetic retinopathy patients and oxygen-induced retinopathy (OIR) mice retina [21]. The inhibition of ADAMTS5 alleviated pathological angiogenesis and upregulated PEDF expression in OIR mice. In addition, ADAMTS5 inhibited PEDF secretion in ARPE-19 cells in in vitro studies and promoted the autophagic degradation of PEDF.

Sex hormones such as oestrogen are contributing factors to diseases such as retinopathy [22]. In a study using rhesus retinal capillary endothelial cells (RhRECs), estradiol induced an increase in the number of viable cells. Cotreatment of oestrogen with phosphatidylinositol 3-kinase (PI3K) and mitogen-activated protein kinase (MAPK) inhibitors significantly decreased the positive effect on cell proliferation and increased PEDF production [23]. Human chorionic gonadotropin (hCG) decreases PEDF expression in ovaries, both in vivo and in vitro, and the expression of PEDF is inverse to that of VEGF [24]. PEDF–VEGF homeostasis is critical to the maintenance of intact ovarian function, and this balance is mediated partly by hCG [25]. The regulation of VEGF and PEDF occurs via protein kinases A and C, as well as epidermal growth factor receptor (EGFR), extracellular signal-regulated kinase 1/2 (ERK1/2), and PI3K [26].

PEDF is highly expressed and secreted in late pregnancy trophoblasts [27]. The removal of PEDF from conditioned medium restored the anti-angiogenic effect from late-pregnancy trophoblasts. PEDF reduced angiogenic network formation only when combined with VEGF, and furthermore, in the chick chorioallantoic membrane (CAM) assay, this combination in fact reduced the branching of vessels. The analysis of the phosphorylation of ERK1/2 and focal adhesion kinase (FAK), two key players in VEGF-induced proliferation and migration, revealed that PEDF altered these VEGF signalling pathways. These data suggest that the trophoblast-derived anti-angiogenic molecule PEDF is involved in restricting the growth and expansion of the foeto-placental endothelium predominantly in late pregnancy and only in tandem with VEGF activity.

In assisted reproductive technologies (ARTs), oocytes are primed to undergo maturation, so they can be extracted and fertilised as required. Usually, this stage is achieved by human chorionic gonadotropin (hCG) triggering (mimicking the midcycle luteinising hormone (LH) surge) [28]. However, the half-life of hCG is considerably longer and spans several days into the luteal phase [29]. In contrast to hCG-triggered mice, OHSS-predisposed mice do not develop OHSS parameters after GnRH-a triggering [30]. GnRH-a triggers a direct effect on PEDF/VEGF balance in granulosa cells inversely to hCG, that is, increased ovarian PEDF to VEGF ratio. Preeclampsia is a hypertensive condition that occurs during pregnancy [31]. VEGF and PEDF were increased in the rat preeclampsia model [32].

PEDF is known to aid the healing of wounds [33] through causing apoptosis-mediated regression of immature blood vessels and stimulation of maturation of the vascular microenvironment, thus promoting a return to tissue homeostasis after injury. In a murine model of skin wound-healing, PEDF was found to be abundant in normal and wounded skin, and was produced predominantly in dermal fibroblasts, bound to microvascular ECs at the injury site, and accumulated in dermal ECM and the epidermis.

Resveratrol (RES) alleviated placental injury and promoted angiogenesis in rats with pre-eclampsia-like symptoms by increasing VEGF via promoting miR-363-3p mediated PEDF suppression [34]. To restrict vascular growth in the retina, Müller glial cells (MGCs) secrete PEDF, which counterbalance proangiogenic factors [35]. PEDF mRNA levels significantly increased in MGCs incubated with RES [36].

In osteogenically differentiated MSCs, PEDF expression increases, as it is a required osteogenic differentiation factor, and possesses anti-osteosarcoma activity with the ability to reduce VEGF levels in cells [37,38,39,40]. To verify whether MSCs promote apoptosis in human umbilical vein ECs (HUVECs), PEDF was silenced via siRNA. The conditioned medium of differentiated MSCs with PEDF silencing significantly improved the proliferation and apoptosis of HUVECs.

Thus, while PEDF has been touted and proven to be a potent antiangiogenic protein, the reality is different, depending on the biological signals at play or the tissue involved. We now realise that it does not only antagonise VEGF activity in the body, but also works together with VEGF in synchronicity to ensure that angiogenesis is properly regulated and ECs are coordinated to fashion patent microvessels as required (detailed in Section 3).

## 3. Using Exogenous PEDF to Curb Angiogenesis in the Eye

For the past two decades, various laboratories have attempted to exploit the anti-angiogenic properties of PEDF towards developing potential treatment strategies. PEDF prevents early mitogenic signals of VEGF-A in primate retinal ECs, inhibiting such physiological events as tube formation [41]. PEDF inhibited the phosphorylation and activation of various downstream VEGF-A signalling partners such as PI3K, AKT (protein kinase B, PKB), and focal adhesion kinase. PEDF bound to the extracellular domain of VEGF-R2, blocking VEGF-A-induced tyrosine phosphorylation of the receptor and inhibiting VEGF-R2 kinase activity.

In mice, an intravitreous injection of the potent anti-angiogenic endostatin into murine retina area resulted in the upregulation of PEDF and downregulation of VEGF, thus restoring the PEDF/VEGF ratio towards antiangiogenesis [42]. Subconjunctival injection with the combination of the clinically used antiangiogenic drug bevacizumab and PEDF in plasmid form successfully inhibited corneal neovascularisation (CNV) in a rat model [43].

PEDF expression attenuated retinal pigment epithelium (RPE) cell proliferation, migration, adhesion, oxidative state, and phagocytic activity with minimal effect on their basal rate of apoptosis [44]. In combination with placental growth factor silencing in RPE cells, PEDF decreases the migration and proliferation of ECs, increases EC apoptosis, and inhibits tube formation [45].

Ocular ischaemia is a typical symptom of diseases that affect the blood vessels of the eye, which includes diabetic retinopathy [46]. Disease-associated changes in the vessels include VEGF overexpression, ocular neovascularisation, and growth of leaky vessels. In an ex vivo ischaemic rat model, PEDF significantly inhibited labyrinth capillary formation and kept the capillary lumen open [47]. The numbers of apoptotic ganglion cells and inner nuclear layer cells were significantly reduced by PEDF.

Ischemic retinopathies are characterised by retinal vasculature impairment and local hypoxia, which cause pathological neovascularisation. In a recent study, found that PEDF-treatment and the PEDF+anti-VEGF (Avastin) treatment groups had a reduced number of apoptosis, and the combination group had the fewest apoptotic cells [48].

High levels of PEDF expression and low levels of VEGF, Fas, and FasL are naturally existent in normal cornea [49]. VEGF levels were significantly induced by chemical cauterisation in the groups treated with chloramphenicol, demonstrating CNV. PEDF prevented cauterisation-induced VEGF overexpression and increased the expression of Fas and FasL. The expression of Fas and FasL correlated to PEDF closely in the development of injury and the response to it.

While there is a purportedly promising efficacy in the treatment of diabetic macular edema (DME) and neovascularisation in diabetic retinopathy (DR) patients via anti-VEGF approaches [50], around 40% of DR patients do not respond [51], signalling the need for other angiogenesis-reducing agents. Furthermore, anti-VEGF treatment has off-target effects, such as retinal fibrosis and neurodegeneration [52]. PEDF34-NP (a poly(lactic-co-glycolic acid) nanoformulation encapsulating the 34mer peptide) reduced ischaemia-induced retinal neovascularisation in an oxygen-induced retinopathy (OIR) rat model, and post-intravitreal administration, significantly reduced retinal vascular leakage and inflammation in diabetic rats [53].

When two optimised peptides (PEDF 335, 8-mer, and PEDF 336, 9-mer) were tested in a mouse model of laser-induced CNV, they both decreased CNV [54]. PEDF 335 also decreased CNV when administered as eye drops. A nanoparticle-conjugate of PEDF 336 had extended efficacy, even when injected 14 days before laser treatment.

Infant retinal neovascularisation in retinopathy of prematurity often regresses spontaneously, but in a majority of patients, warrants management by laser ablation or local VEGF antagonism [55]. Approaches involving anti-VEGF may cause systemic suppression of normal angiogenesis, leading to improper organ development [56]. This same team then demonstrated PEDF 336 in a newborn mouse OIR model, showing that angiogenesis was reduced in a dose-responsive manner by single intravitreal injections more dose-effectively than anti-mouse VEGFA164 [57]. Intriguingly, the combination of anti-VEGFA164 with PEDF 336 gave only the poorer anti-VEGF response while abrogating the robust inhibition seen with peptide alone. The authors [57] proposed that there was a need for VEGF in priming the endothelium to the peptide.

Co-MSC secretome significantly inhibits tube and sprout formation in HUVECs [58]. Co-MSCs from different donors consistently contained high levels of PEDF and low levels of VEGF-A. The application of co-MSCs to mouse corneas after injury prevented the development of CNV. Stripping the secretome off PEDF diminished the antiangiogenic effects of co-MSCs. Co-MSCs isolated from PEDF KO mice had reduced antiangiogenic potential.

Thus, the above studies, albeit leaning heavily towards ocular indications, promisingly show that the administration of PEDF or even its peptides can assist in angiogenesis-mediated improvements in disease conditions, particularly of the eye (at the retina or cornea). Now, this review will discuss the use of PEDF or its active peptides as anti-angiogenic agents in other organs.

## 4. Administration of PEDF for Dampening of Angiogenesis in Other Conditions

As introduced above, the use of PEDF to inhibit angiogenesis for therapeutic benefit is not limited to the eye or its ailments. inhibits the phosphorylation and activation of VEGF-A signalling partners ((PI3K, AKT, FAK, Src (Y416), and phospholipase C gamma (PLC-γ)) [41]. It binds to the extracellular domain of VEGF-R2, blocking VEGF-A-induced phosphorylation of Tyr 951 and Tyr 1175, and inhibiting VEGF-R2 kinase activity. PEDF has no effect on the transcription or translation of VEGF-R2 in HUVECs, and it also can bind to the extracellular domain of VEGF-R1. In osteosarcoma cells, PEDF is known to cause the downregulation of VEGF-A [39], and its peptide can do the same [59]. PEDF decreases vasculature in adipose tissue when administered locally [60].

The P18 PEDF peptide targets VEGFR2 on ECs and, by doing so, it modulates signalling transduction between VEGF/VEGFR2 and suppresses activation of PI3K/Akt cascades, leading to an increase in mitochondrial-mediated apoptosis and anti-angiogenic activity [61]. The anti-VEGF effect of a 7-mer (based off the 34-mer PEDF peptide) was 1.5-fold greater than that of the 34-mer [62]. Significant apoptosis (37%) was induced by 7-mer treatment in multiple myeloma cells. Apoptosis and apoptosis-associated proteins induced by the 7-mer were blocked by the inhibition of c-Jun N-terminal kinase (JNK), but not p38 MAPK. The 7-mer inhibited VEGF-mediated angiogenesis of ECs in vitro, and CNV in mice. G24, a PEDF-based peptide, significantly inhibited proliferation in VEGF-activated human umbilical vein ECs (HUVECs) [63]. G24 exhibited strong inducing effects on cell apoptosis and internalisation of 67LR (one of the receptors for PEDF, laminin receptor), and inhibited cell migration and angiogenesis in vitro.

PEDF inhibits the increase of endothelial permeability, akin to the PEDF receptor inhibitor Atglistatin [64]. PEDF also dampens the inhibitory effect of damaged ECs on haematopoietic stem cell expansion in vitro. Additionally, PEDF accelerates haematopoietic reconstitution after bone marrow transplantation in mice. PEDF could induce HUVEC autophagy by sequentially inducing p53 and sestrin2 expression [65]. The authors proposed inhibition of the mechanistic target of rapamycin kinase to be the mechanism responsible for PEDF–induced autophagy; as p70S6K and 4E-BP1 phosphorylation levels were significantly upregulated in cells knocked down for p53 and sestrin2.

The PEDF gene delivered by cyclic arginylglycylaspartic acid–polyethyleneglycol–polyetherimide suppressed tumour growth and decreased MVD in nude mice bearing human colorectal xenografts [66]. The cRGD moiety boosted the transfection efficiency of PEG–PEI in human umbilical vein endothelial cells (HUVECs). In a study comparing WT-PEDF with a phosphomimetic mutant called EEE-PEDF, both peptides inhibited cancer cell motility, with EEE-PEDF showing a stronger effect [67]. The more potent activity of EEE-PEDF was correlated to a better binding of LR67. Furthermore, the proapoptotic and antimigratory activities were found to be respectively regulated by differential activation of two distinct MAPK pathways, namely JNK and p38. JNK leads to apoptosis in ECs, while p38 leads to antimigratory effects in both cancer cells and ECs.

In the endometrial environment, PEDF expression is subjected to regulation by steroid hormones, both in vivo and in vitro [68]. Oestradiol decreases PEDF expression and progesterone increases its levels. In clinical endometrial specimens, PEDF levels were dynamically altered throughout the menstrual cycle, being understandably low at the proliferative and early secretory phases and significantly higher at the late-stage secretory phase. The expression levels of PEDF were inversely correlated with VEGF. In addition, oestradiol decreases and progesterone increases PEDF secretion into conditioned media by both normal endometrial stromal fibroblasts and cancer-associated fibroblasts, but only conditioned medium from endometrial stromal fibroblasts inhibited the growth of primary endometrial epithelial cells [69].

Pegylated nanoparticles encapsulating PEDF exerted antiangiogenic effects on high glucose-stimulated HUVECs and showed promise in alleviating microvascular dysfunction [70]. These effects included attenuated HUVEC proliferation, migration, tube formation, plus dampened VEGF secretion. In cultured human dermal microvascular endothelial cells, PEDF alone or in combination with inflammatory mediators results in the activation of RhoA, impairing endothelial barrier function and causing leakage during sepsis [71]. PEDF-induced hyperpermeability was mediated through the ATGL receptor.

## 5. PEDF and Diabetes

The diabetic link of PEDF is a much more recent one compared with its link to angiogenesis. There have been some defining clinical studies that led research back to the lab and preclinical models to better understand the true relationship of PEDF with this chronic illness. Suffice it to say, the situation is not as dire as originally thought.

Higher PEDF concentrations have been observed in patients with diabetes relative to healthy controls, especially in those with vascular complications, compared with patients without vascular disease [72,73]. In diabetes-associated chronic kidney disease (CKD) condition, there are positive associations between PEDF and CKD progression, though a causal link between PEDF and CKD has not been proven [74]. Body mass index, triglycerides, and fasting C-peptide were positively associated with the serum PEDF level, and the high-density lipoprotein cholesterol and estimated glomerular filtration rate were inversely associated with the serum PEDF level [75].

Elevated circulating levels of PEDF are found in patients with type 2 diabetes, and the factor is associated with the development and progression of CKD type 2 diabetics [76]. Participants with de novo sight-threatening diabetic retinopathy had significantly higher baseline PEDF levels in the serum [77]. However, renal PEDF levels were significantly reduced in genetic models of type 1 and type 2 diabetes (Akita and db/db, respectively) [78]. The PEDF levels were negatively correlated with renal Wnt signalling activity and was shown to inhibit Wnt-mediated fibrosis in renal proximal tubule epithelial cells. This was further confirmed in a PEDF KO model in which mice exhibited renal fibrosis, oxidative stress, inflammation, and renal tubule epithelial cell apoptosis. So, there seems to be a discrepancy between PEDF levels inside and outside of tissues, such as that in the kidney in relation to diabetes.

A small bioactive PEDF peptide (P78-PEDF; amino acids 78–121) prevented the development of diabetic nephropathy (DN) [79]. P78 peptide treatment not only prevented the development, but also the progression, of DN in the Ins2Akita murine diabetic model, as indicated by decreased albuminuria, decreased kidney macrophage recruitment, and inflammatory cytokines (kidney TNF-α, fibronectin, VEGFA and EGFR), reduced histological changes, and increased nephrin expression. The peptide (but not the clinically used drug captopril) restored nephrin and reduced kidney VEGFA and EGFR expressions.

Diabetic nephropathy (DN) is the most severe complication of diabetes worldwide and is the leading cause of end-stage kidney disease [80]. Treatment with P78-PEDF protected diabetic mice from DN displaying reduced albuminuria, kidney macrophage recruitment, histological changes, inflammatory cytokines, and fibrotic markers (kidney TNF-α, fibronectin, VEGFA, and EGFR), and restoration of nephrin expression [81].

PEDF is located in the kidney glomeruli, and its expression in the kidneys of diabetic mice declines significantly [82]. Continuous infusion of P78-PEDF stabilises kidney disease by reducing blood urea nitrogen, serum creatinine, renal macrophage recruitment, inflammatory cytokines, and histological changes, and restores TLR4/NF-κB signalling in diabetic mice. PEDF blocks the advanced glycation end-product-induced apoptotic cell death of podocytes by suppressing RAGE expression and subsequent ROS generation, partly via PPARγ activation [83].

Elevated circulating levels of PEDF were also found in Type 2 diabetic versus non-diabetic subjects [84], and in Type 1 diabetic subjects with versus those without microvascular complications [72]. In a study group of mostly male US veterans (average age 62 years), PEDF was positively associated with serum triglycerides, waist-to-hip ratio, serum creatinine, use of ACE inhibitors or angiotensin receptor blockers, and use of lipid-lowering agents [85].

Diabetic wounds exhibited significant perturbations in the expression of factors that affect vascular regrowth, maturation, and stability, and this includes the downregulation of PEDF [86]. Hyperglycaemia increases retinal oxidation and is also associated with abnormal vascular changes, such as dilatation and deformation, with increased levels of PEDF [87]. Again, this aligns with the inside tissue versus outside (blood circulation) scenario mentioned above.

PEDF KO mice displayed increased adiposity, glucose intolerance, and elevated serum levels of metabolites, pointing towards metabolic syndrome [88]. The livers of PEDF KO mice showed impaired IRS and Akt signalling. When human hepatocytes were challenged with IL-1b, PEDF blocked the challenge by suppressing the activation of c-Jun N-terminal kinase while restoring Akt signalling. A metabolomics profile identified elevated circulating levels of tricarboxyclic acid cycle intermediates, including succinate, an inducer of IL-1b, in PEDF KO mice. Succinate-dependent IL-1b expression was blocked by PEDF in PEDF KO hepatocytes. In PEDF KO mice, PEDF administration reduced hyperglycaemia and improved hepatic insulin signalling.

PEDF influences glucose homeostasis from the inhibition of the insulin receptor to the effector phosphorylation through Akt/PKB-dependent and -independent pathways in human and murine skeletal muscle cell lines [89]. The exposure of skeletal myocytes to PEDF attenuates insulin-dependent insulin receptor autophosphorylation, tyrosine phosphorylation of insulin receptor substrate 1, and dual loop phosphorylation–activation of Akt. PEDF switched off both the molecular inducers of glucose transporter 4 (GLUT4) translocation: IRS-Akt/PKB-AS160-mediated and IR-pCbl-dependent GLUT4 translocation. This turned the notion that PEDF contributes to metabolic syndrome [90] on its head.

Furthermore, PEDF inhibits the phosphorylation of insulin receptor (IR) and insulin receptor substrate (IRS) in skeletal myocytes [91]. PEDF constitutively activates p42/44 MAPK/Erk, but paradoxically does not affect mitogenic signalling. PEDF increased chronic and acute insulin secretion in a clonal rat β-cell line without alteration of glucose consumption [92]. PEDF stimulates insulin secretion from primary mouse islets without changing mitochondrial respiration and glycolytic function. PEDF enhances ATGL-mediated stimulation of glycerol release, with an increase in intracellular ATP levels. All the above studies point to PEDF being beneficial in diabetes.

Taking the next step, in our lab, we found that PEDF activates Erk1/2 MAPK signalling, which is crucial for PEDF-mediated osteogenesis [93]. Insulin was found to block this pathway, thus inhibiting PEDF-induced osteogenic differentiation of skeletal muscle cells. In the future, the use of PEDF-incorporated muscle grafts and flaps could be employed to accelerate bone regeneration, with such heterotopic ossification controlled via the co-administration of insulin.

Diabetic cardiomyopathy (DCM), in the absence of hypertension and coronary artery disease, but stemming from the diabetic condition, is characterised by structural remodelling and deterioration of cardiac function, which may lead to heart failure and mortality if unchecked [94]. PEDF overexpression reversed cardiac remodelling in db/db mice, with modulation of cardiac energy metabolism, reduction of lipotoxicity, and increased ATGL and glucose transporter type 4 (Glut4) levels [95]. PEDF also reduced the expression of peroxisome proliferator-activated receptor alpha (PPARγ), carnitine palmitoyltransferase 1 alpha (CPT1α), and scavenger receptor B2 (CD36). It also reduced ROS generation and apoptosis in db/db mice myocardium.

## 6. PEDF and Metabolism

PEDF is one of the most abundant proteins secreted during adipocyte maturation directly linked to central obesity [96,97,98,99]. The commentary around the 2008–2015 era was that PEDF was a causative agent for metabolic disorders. However, with continued efforts in various labs, including ours, that view has pretty much been put to rest. PEDF is more thought of now as a preventative or protective protein trying to subvert these dysfunctional metabolic states.

PEDF positively correlates with BMI and declines when healthy individuals are challenged with an oral glucose load [100]. PEDF concentrations are similar in obese children, regardless of type 2 diabetes (T2D) status, and higher in obese compared with normal-weight children, regardless of diabetes [101]. Following a low-calorie diet regimen, rheumatoid arthritis (RA) with low-moderate disease activity reaching BMI reduction ≥5% at 6 months correlated with a decrease in plasma PEDF, in parallel with an improvement in disease [102].

In a fasting blood glucose test, PEDF-null mice had glucose levels that were lower by ~31% at 15 weeks of age compared with wildtype mice, representing an approximately 22% overall decrease in glucose levels with increasing age in PEDF-null mice compared with age-matched wildtype controls [103].

The expression levels of PEDF decreased by 50% in interscapular brown adipose tissue (iBAT) from day 2 onwards of cold exposure [104]. In contrast, for subcutaneous white adipose tissue (sWAT) and epididymal white adipose tissue (eWAT), cold exposure only induced transient increases at day 4 and day 1, respectively. In vitro, PEDF was transiently increased in both brown and white adipocytes treated with a β3-adrenoceptor agonist.

Psoriatic arthritis (PsA) is a musculoskeletal disease affecting multiple parts of the body, and is usually accompanied by other comorbidities, such as metabolic syndrome [105]. Some patients with PsA may exhibit axial involvement, referred to as axial PsA, while those without axial joint involvement are known as peripheral PsA. In axial psoriatic arthritis patients, PEDF correlates positively with BMI and C-reactive protein [106]. 

In hepatocellular carcinoma, intracellular PEDF is noted to be precancerous, while extracellular PEDF is anticancerous [107]. The intracellular PEDF causes the accumulation of free fatty acids (FFAs) in vivo and in cultured tumour cells. When human primary normal prostate fibroblasts were compared with cancer associated fibroblasts (CAFs) for lipid content, triacylglycerol-regulating proteins, and microtubule-organising centre number and distribution, CAFS stored more neutral lipids and had minimal or absent PEDF and ATGL protein levels [108].

What happens when PEDF or its peptides are used against metabolic disorders? PEDF administration to lean mice induces insulin resistance, whereas PEDF neutralisation in obese rodents enhances insulin sensitivity [90]. PEDF levels are elevated in subjects with metabolic syndrome [109] and type 2 diabetes [110]. In a very-low-calorie-diet study, plasma levels of PEDF decreased between the baseline and week 8, and increased between weeks 16 and 18 [111]. In the fast food study, the plasma levels of PEDF increased between the baseline and week 4.

Using rat adipose-derived stem cells and the mouse pre-adipocyte cell line 3T3-L1, knockdown of PEDF in differentiating cells resulted in elevated levels of ATGL and CD36 (regulates proliferation and lipogenic gene expression during adipogenesis), as well as other adipogenic markers, increasing adipocyte number [112]. PEDF expression was regulated by dexamethasone, a glucocorticoid widely used for adipogenesis at the transcriptional level.

Non-alcoholic fatty liver disease (NAFLD) is characterised by the accumulation of lipids in the liver and covers a wide spectrum of liver disorders, ranging from benign simple steatosis to steatohepatitis (NASH) and advanced fibrosis and cirrhosis [112]. NAFLD is prevalent in developed countries, and is commonly accompanied by metabolic syndrome, which includes dyslipidaemia, obesity, and type 2 diabetes [113]. Its incidence of NAFLD is on a sharp rise due to the obesity epidemic. It has been suggested [114] that PEDF downregulation in the liver leads to triglyceride accumulation, which may progress to NAFLD.

Lipid droplets (LDs) are cytoplasmic organelles consisting of a core of neutral lipids surrounded by a phospholipid monolayer with proteins either embedded in this monolayer or attached to its surface [115]. Intracellular lipid homeostasis is maintained by specific proteins, which regulate the balance between lipolysis and lipogenesis. Many of these proteins are located on the surface of LDs. One of these is PEDF, which stimulates lipolysis and the release of free fatty acids (FFAs), as noted above. Highly aggressive prostate, breast, and pancreatic cell lines had significantly higher maximum LD velocity (LDVmax) than less aggressive and benign cells [116]. LDVmax was microtubule-dependent and suppressed by blocking V-ATPase or exposure of cells to PEDF. A link between LDV and metastasis was proposed.

When isolated neonatal and adult left-ventricular cardiomyocytes were subjected to oxygen–glucose deprivation (OGD), PEDF significantly reduced AMP-activated protein kinase alpha (AMPKα) levels to decrease ATP production and reduced ATP expenditure [117]. This essentially increased energy reserves and cell viability. AMPKα was degraded by a ubiquitin-dependent proteasomal degradation pathway, linked to a PEDF/PEDFR/peroxisome proliferator activated receptor γ (PPARγ) axis. PPARγ or proteasome inhibition both disrupted the interaction of AMPKα and PPARγ, thus cancelling AMPKα degradation.

In HFD mice, PEDF effectively decreased body weight gain, white adipose tissue mass, and inflammation, and improved insulin resistance, dyslipidaemia, and hyperglycaemia [118]. In liver tissue, PEDF decreased lipid accumulation and fibrosis. In a murine model of nonalcoholic steatohepatitis, PEDF could slow the development and progression of steatohepatitis through the suppression of steatosis and inflammatory response [119]. The diet-induced upregulation of NADPH oxidase components was dampened in PEDF-treated mice.

The fact that one of PEDF’s receptors, ATGL, functions to regulate lipids in cells aligns well with PEDF’s link to various metabolic disorders. Given the growing body of work linking various diseases to metabolic underlying causes, the implication for PEDF is not small. The initial negative sentiment in the early 2000s towards this protein being a causative agent for various metabolic disorders has given way to a better understanding that it is perhaps upregulated as a protective mechanism to control these disorders from spiralling out of control. However, we have just scratched the surface in this field, and much more needs to be done.

## 7. PEDF and Cardiovascular Disease/Disorders (CVDs)

The link between PEDF and CVD is not discussed as much as its link with eye disease centred around angiogenesis or its activities against various types of cancers. However, as this paper points out below, there are several impactful findings that have been reported that further add versatility to this potent protein. One of the definitive studies on the CVD and PEDF link was that, when apolipoprotein E KO/PEDF KO mice were fed with 24, 36, or 48 weeks of high-fat diet, greater accumulation of body fat and plasma lipids were displayed in PEDF-deficient mice [120]. Importantly, PEDF deficiency in mice accelerated atherosclerosis, as seen by increased atherosclerotic plaques, pronounced vascular dysfunction, and increased lipid accumulation in peripheral tissues, whereas intravenous injection of adeno-associated virus transducing PEDF achieved the opposite.

PEDF overexpression improves atherosclerotic plaque stability in apolipoprotein E KO mice [121]. The expression of inflammatory factors (interleukin-1β, interleukin-6, tumour necrosis factor-α, monocyte chemotactic protein-1, and matrix metalloproteinase) was decreased when PEDF was overexpressed. The activity was mediated through PPAR-γ. The serum PEDF level is a significant independent predictor associated with necrotic core progression during statin therapy, and its levels could be elevated as a counter-regulatory response mechanism (akin to what happens in obesity) to protect against necrotic core progression [122].

PEDF may be associated with acute coronary syndrome, and at least regions of possible polymorphisms of PEDF are correlated with CVD [123]. OxLDL decreased PEDF concentrations by increasing ROS. When PEDF is not allowed to be downregulated, ECs are protected from the harmful effects of oxLDL. Furthermore, PEDF might alleviate endothelial injury by inhibiting the Wnt/β-catenin pathway. Indeed, oxLDL-induced HUVEC injury and apoptosis, oxidative stress, and activation of the Wnt/β-catenin pathway can all be suppressed by PEDF [124].

In isolated rat neonatal and adult left-ventricular cardiomyocytes subjected to OGD, PEDF significantly decreased the AMPKα levels to decrease ATP production and decrease ATP expenditure both in neonatal and adult cardiomyocytes, which increased energy reserves and cell viability [117]. Under hypoxia, PEDF promoted the inhibition of p53 mitochondrial translocation via PEDF-R in cardiomyocytes [125]. Consequentially, mitochondrial outer-membrane permeabilisation and mitochondrial permeability transition pore opening were both inhibited, and apoptosis and necrosis were affected.

PEDF increases the number of autophagosomes, increases the viability and decreases cleavage of caspase 3 in H9c2 cells facing hypoxia [126]. However, in cardiomyocytes derived from neonatal mice, PEDF increased cardiomyocyte apoptosis during hypoxia via Fas mediated through receptor PLA2 [127]. PEDF bound to PEDF-R expressed on the membrane of H9c2 cells, and under hypoxia, inhibited p53 mitochondrial translocation via PEDF-R [125]. As a result, mitochondrial outer membrane permeabilisation (MOMP) and mitochondrial permeability transition pore (MPTP) opening were inhibited, and cleaved caspase-3, PARP and the release of high mobility group box 1 were decreased. Thus, apoptosis and necrosis were attenuated.

Apoptosis and necroptosis are significantly increased after hypoxia in cultured H9c2 cells and primary cardiomyocytes [128]. Both PEDF and the 44-mer reduced apoptosis and necroptosis. PEDF and the 44-mer upregulated super oxide dismutase, catalase, and glutathione peroxidase levels, thus reducing the levels of ROS and malondialdehyde in these cells. In hypoxic primary neonatal cardiomyocytes, PEDF decreases mitochondrial density through promoting hypoxic cardiomyocyte mitophagy [129]. Under OGD conditions, PEDF and its peptide 44-mer reduce OGD-induced oxidative stress via its receptor PEDF-R and the PPARγ signalling pathway in H9c2 cells, thereby reducing apoptosis [130].

In neonatal cardiomyocytes, PEDF decreased the activation of the nucleotide-binding oligomerisation domain-like receptor protein 3 inflammasome [131]. It reduced dynamin-related peptide 1-induced mitochondrial fission, and in turn, mitochondrial fission-induced mitochondrial DNA and mitochondrial ROS release into cytosol through PEDFR/iPLA2 (calcium-independent phospholipase A2).

PEDF decreased the levels of ROS and malondialdehyde in hypoxia/regeneration-conditioned H9c2 cells [132]. When challenged with hypoxia/regeneration, PEDF decreased ROS in the mitochondria, increased the mitochondrial DNA copy number, and decreased xanthine oxidase and nicotinamide adenine dinucleotide phosphate oxidase activity, as well as RAC family small GTPase 1 protein expression.

Apart from dampening ROS, PEDF directly increases glucose uptake in hypoxic cardiomyocytes [133]. The levels of GLUT4 at the plasma membrane of hypoxic cardiomyocytes were increased in the presence of PEDF, but its total amount was not changed. The cardioprotective effects induced by PEDF were blocked by GLUT4 inhibition. The PEDF-mediated GLUT4 translocation and glucose uptake increase in hypoxic cardiomyocytes were prevented by PI3K or AKT inhibition.

In an acute myocardial infarction model, PEDF significantly reduced the size of the infarcted myocardium [134]. PEDF significantly upregulated PI3K-AKT permeability and the distribution of ZO-1 between ECs during OGD. PEDF effectively reduced the infarction area and improved cardiac function in AMI rats [135]. Furthermore, PEDF upregulated the expression of tight junction proteins in vivo and in vitro. Mechanistically, PEDF inhibited the expression of phosphorylated low-density lipoprotein receptor-related protein 6 and active β-catenin under OGD, thereby suppressing the activation of the classical Wnt pathway.

PEDF not only reduced myocardial infarct size, but also improved cardiac function in rats with acute myocardial infarction (AMI) [136]. Thus, PEDF may protect cardiac function from ischemic injury by reducing vascular permeability, cardiomyocyte apoptosis, and myocardial infarct size. In an AMI model, PEDF increased the native collateral blood flow and significantly inhibited its decline [137]. This improves cardiac function and induces ventricular remodelling. The overexpression of PEDF in young MSCs impaired the beneficial effects against MI injury and induced changes in the infarct region, similar to when old MSCs were administered [138]. These changes include fewer ECs, vascular smooth muscle cells, and macrophages, but more fibroblasts, as PEDF is known to increase cardiac fibrosis. When PEDF was expressed through intramyocardial gene delivery 5 days before AMI/recanalisation in rats, PEDF was found to boost microvascular reperfusion 4 h postcoronary occlusion [139]. PEDF maintained the stability of endothelial adherens junctions, maintaining endothelial junctions, and preventing the occurrence of no-reflow.

Post-MI, the maintenance of myocyte contractility, by increasing Ca2+ influx, exacerbates cardiac injury and pump dysfunction by decreasing the myocyte number [140]. In a rat AMI model, PEDF local overexpression enhanced cardiac functional reserve and reduced myocardial contracture around the edge of the infarcted area [141]. 

Thus, the above studies link PEDF with CVD disease, and this is not that surprising given PEDF’s role in EC biology. In the future, armed with this knowledge, it is foreseeable that PEDF or its peptides may be used therapeutically post-clinical evaluation in varying models of CVD.

## 8. Future Directions

**Figure 1:**
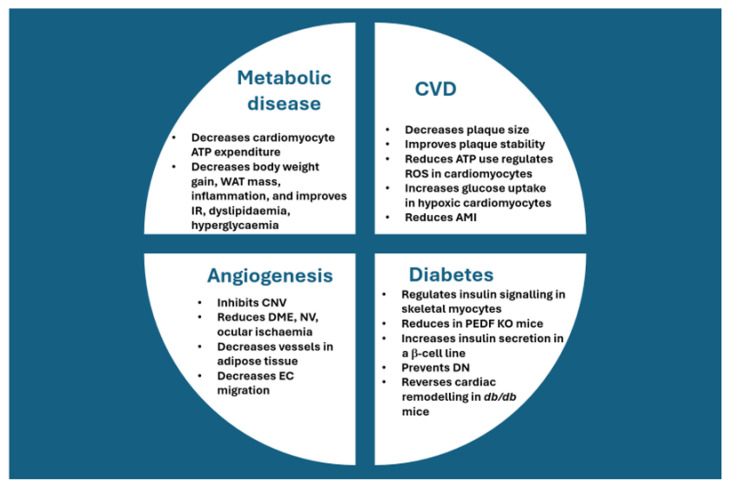
The effect of exogenous PEDF administered in cases of angiogenesis, cardiovascular disease, diabetes, and metabolic diseases. Abbreviations: AMI, acute myocardial infarction; ATP, adenosine triphosphate; CNV, corneal neovascularisation; DME, diabetic macular oedema; DN, diabetic nephropathy; EC, endothelial cell; HFD; high-fat diet; KO, knockout; NV, neovascularisation, PEDF, pigment epithelium-derived factor; ROS, reactive oxygen species; WAT, white adipose tissue.

The litmus test for PEDF will be the ability to produce the protein on a large scale in a cost-effective manner to allow clinical utility against the four disease indications discussed in this paper (summarised in Figure 1). To this end, stably transfected human embryonic kidney cells were utilised to produce recombinant PEDF and further purified via ion-exchange column chromatography [142]. Milligram amounts of highly purified protein were recovered and found to bind with high affinity to PEDFR. No biological studies have been performed to evaluate the activity/functionality of the protein, but it is heartening to PEDF researchers that cheaper alternative sources are available to produce milligram quantities of the serpin for studies.

The amino acid sequence of PEDF has been altered to increase its affinity for heparin and hyaluronic acid, both negatively-charged ECM molecules [143]. This engineered PEDF had higher affinity for heparin and HA than wild-type PEDF. Engineered PEDF exhibited antiangiogenic and retinal survival bioactivities. It also inhibited EC proliferation and tube formation in vitro, and protected photoreceptors from cell death in an ex vivo model mimicking retinal degeneration.

While PEDF constitutively activates p42/44 MAPK/Erk, it does not affect mitogenic signalling, which is a great outcome [91]. PEDF does not perturb neither mitochondrial activity nor proliferation in cells such as mesenchymal stem cells, cardiomyocytes, and skeletal myocytes and adipocytes. In our lab, the PEDF protein has been encapsulated within microparticles, and this has been found to be bioactive in a bone formation model in vivo [144] and anticancerous against bone cancer [145]. Plasma transfusion (with natural levels of PEDF) into PEDF −/− type VI osteogenesis imperfecta individuals was conducted, but effects were not seen due to the low levels of PEDF administered, the low duration of dosing, and the low numbers of patients evaluated [146]. However, this is a groundbreaking study. Perhaps microparticle delivery [147] or the use of hydrogel [148], which has been trialled for PEDF gene delivery, may achieve better biological effects.

Moving away from recombinant protein, perhaps plasmid or viral gene delivery may produce better results. Indeed, we have demonstrated that plasmid DNA was efficacious in spontaneously metastasising a model of osteosarcoma in vivo [145]. Indeed, liposomal nanoparticles are still being formulated and tested, with varying degrees of success in vivo [149].

As the 34-mer PEDF interacts with different PEDF receptors, it can be used to design peptides for future therapeutic aims [59,150]. This may be the way to go, as the full-length protein has various functions, and some may interfere with the intended therapeutic use. The use of the 34-mer and 44-mer has shown us that more streamlined effects can be derived from the use of short peptides (reviewed in [151]).

The protein is safe in the eye, as no side-effects were found in rat eyes after intravitreal PEDF protein administration [152]. We have found the protein to be quite stable and easily dissolved in water or buffered saline [37]. While PEDF can be used to regenerate damaged disks of the vertebrae by slowing down cell death [153], it is not critical for haematopoietic stem cell regeneration [154]. MSCs can be induced to form de novo bone tissue in vivo [148]. In our lab, we have demonstrated that PEDF can induce bone formation in vivo via transdifferentiating either muscle [93,144] or adipocytes [91].

Thus, there are various parallel lines of investigations for PEDF, but yet, we await a clinical trial for the protein. The cost of making the protein is high, as prices have increased exponentially over the past two decades, limiting and perhaps even inhibiting certain forms of research, particularly in vivo, where milligram quantities of protein may be needed for effective evaluation. It is this authors view that it will be short peptides, not the full-length protein, that will eventually reach and prove successful in clinical trials.

## Abbreviations

ADAMTS5	A disintegrin and metalloproteinase with thrombospondin motifs 5
PKB	Protein kinase B
AMI	Acute myocardial infarction
AMPK	Adenosine monophosphate-activated protein kinase
ATGL	Adipose triglyceride lipase
CKD	Chronic kidney disease
CVD	Cardiovascular disease
DN	Diabetic nephropathy
DR	Diabetic retinopathy
EC	Endothelial cell
ECM	Extracellular matrix
EGFR	Epidermal growth factor receptor
ERK	Extracellular signal-regulated kinase
GLUT	Glucose transporter
Hcg	Human chorionic gonadotropin
HUVEC	Human umbilical vein EC
Inos	Inducible nitric oxide synthase
IRS	Insulin receptor substrate
JNK	c-Jun N-terminal kinase
KO	Knockout
MAPK	Mitogen-activated protein kinase
MVD	Microvessel density
MGC	Müller glial cell
MSC	Mesenchymal stem cell
OGD	Oxygen-glucose deprivation
OHSS	Ovarian hyperstimulation syndrome
OIR	Oxygen-induced retinopathy
OxLDL	Oxidised low-density lipoprotein
P13K	Phosphatidylinositol 3-kinase
PEDF	Pigment epithelium-derived factor
PPAR-γ	Peroxisome proliferator-activated receptor gamma
RES	Resveratrol
ROS	Reactive oxygen species
RPE	Retinal pigment epithelium
SERPIN	Serine protease inhibitor
Sev	Small extracellular vesicle
SSc	Systemic sclerosis
TGF-β	Transforming growth factor β
VCAM-1	Vascular cell adhesion molecule-1
VEGF	Vascular endothelial growth factor receptor
Wnt	Wingless-related integration site
